# ANALYSIS OF FOOD TOLERANCE IN PATIENTS SUBMITTED TO BARIATRIC SURGERY USING THE QUESTIONNAIRE QUALITY OF ALIMENTATION

**DOI:** 10.1590/S0102-6720201500S100021

**Published:** 2015-12

**Authors:** Matheo Augusto Morandi STUMPF, Marcos Ricardo da Silva RODRIGUES, Ana Claudia Garabeli Cavalli KLUTHCOVSKY, Fabiana TRAVALINI, Fábio Quirillo MILLÉO

**Affiliations:** Department of Medicine, State University of Ponta Grossa, Ponta Grossa, PR, Brazil

**Keywords:** Bariatric surgery, Alimentation, Questionnaires

## Abstract

***Background*:**

Due to the increased prevalence of obesity in many countries, the number of bariatric surgeries is increasing. They are considered the most effective treatment for obesity. In the postoperative there may be difficulties with the quality of alimentation, tolerance to various types of food, as well as vomiting and regurgitation. Few surveys are available to assess these difficulties in the postoperative.

***Aim*:**

To perform a systematic literature review about food tolerance in patients undergoing bariatric surgery using the questionnaire "Quality of Alimentation", and compare the results between different techniques.

***Method*:**

A descriptive-exploratory study where the portals Medline and Scielo were used. The following headings were used in english, spanish and portuguese: quality of alimentation, bariatric surgery and food tolerance. A total of 88 references were found, 14 used the questionnaire "Quality of Alimentation" and were selected.

***Results*:**

In total, 2745 patients were interviewed of which 371 underwent to gastric banding, 1006 to sleeve gastrectomy, 1113 to Roux-en-Y gastric bypass, 14 to biliopancreatic diversion associated with duodenal switch, 83 were non-operated obese, and 158 non-obese patients. The questionnaire showed good acceptability. The biliopancreatic diversion with duodenal switch had the best food tolerance in the postoperative when compared to other techniques, but it was evaluated in a single article with a small sample. The longer the time after the operation, the better is the food tolerance. Comparing the sleeve gastrectomy and the Roux-en-Y gastric bypass, there are still controversial results in the literature. The gastric banding had the worst score of food tolerance among all the techniques evaluated.

**Conclusion:**

The questionnaire is easy and fast to assess the food tolerance in patients after bariatric surgery. Biliopancreatic diversion with duodenal switch had the best food tolerance in the postoperative when compared to sleeve gastrectomy and the Roux-en-Y gastric bypass. Gastric banding still remains in controversy, due it presented the worst score.

## Abstract (pt)

***Introdução*:**

Devido ao aumento da prevalência da obesidade em diversos países, o número de operações bariátricas está aumentando. Elas são consideradas o tratamento de maior eficácia da obesidade. No pós-operatório podem ocorrer dificuldades com relação à qualidade da alimentação, tolerância para vários tipos de alimentos, além de vômito e regurgitação. Poucos questionários estão disponíveis para avaliar essas dificuldades no pós-operatório *.*

***Objetivo*:**

Realizar revisão sistemática da literatura sobre a tolerância alimentar em pacientes submetidos à cirurgia bariátrica utilizando o questionário "Quality of Alimentation" e comparar os resultados entre as diferentes técnicas.

***Método*:**

Estudo descritivo-exploratório onde foram pesquisados os portais de periódicos Medline e Scielo. Utilizaram-se os descritores "quality of alimentation, bariatric surgery e food tolerance", em inglês, português e espanhol. Foram encontradas 88 referências, sendo 14 selecionadas por utilizarem o questionário "Quality of Alimentation".

Ao total, 2745 pacientes foram entrevistados sendo que 371 foram submetidos à bandagem gástrica, 1006 à gastrectomia vertical, 1113 ao bypass gástrico em Y-de-Roux, 14 à derivação biliopancreática associada ao duodenal switch, 83 eram obesos não operados, e 158 pacientes não obesos. O questionário teve boa aceitação. A derivação biliopancreática com duodenal switch apresentou a melhor tolerância alimentar no pós-operatório quando comparada às demais técnicas, mas foi avaliada em um único artigo e com pequena amostra. Quanto maior o tempo de pós-operatório, melhor foi a tolerância alimentar. Comparando a gastrectomia vertical e o bypass gástrico em Y-de-Roux, ainda há resultados controversos na literatura. A banda gástrica apresentou o pior escore de tolerância alimentar dentre todas as técnicas avaliadas.

**Conclusão:**

: O questionário é de fácil e rápida aplicação para avaliar a tolerância alimentar em pacientes submetidos à cirurgia bariátrica. A derivação biliopancreática com duodenal switch teve a melhor tolerância alimentar no pós-operatório quando comparada à gastrectomia vertical e ao bypass gástrico em Y-de-Roux. Quanto à banda gástrica ainda existe controvérsia quanto ao seu uso, devido à apresentação de piores resultados.

## INTRODUCTION

Currently obesity is highly prevalent in several countries. Brazil, in 2012, was estimated to have 17.2% of obese adults^12^. As the worldwide prevalence, the World Health Organization estimates that in 2015 there are approximately 2300 million adults overweight and over 700 million obese^21^.

Among the possible treatments is the pharmacological approach. The most commonly used agents are: β-fenetilaminic derivatives, tricyclic, fenilpropanolamic, oxitrifluorfenil phenylpropanolamine, naftilaminic and derived from lipstatine^10^. The major problem of these treatments are the possible side effects and do not result in major weight loss. Currently it is believed that one of the most effective treatments to combat obesity is the bariatric surgery. However, the operation is indicated only for patients with a body mass index (BMI) above 40 kg/m^2^ or a BMI between 35 and 39.9 kg/m^2^ with associated comorbidities. The clinical treatment failure for more than two years is also necessary for indication^22^.

Traditionally, postoperative results in bariatric surgery are valued by BAROS (Bariatric Analysis and Reporting Outcome System). Oria and Moorhead^14^ proposed this questionnaire, where the score is based on the loss of excess weight, improvement in comorbidities and quality of life, and post-surgical complications. Depending on the score is defined if the patient has succeeded or failed operation. However, does not evaluate the food tolerance, which is an important factor in postoperative the different techniques used^13^. Until now, purely restrictive (such as laparoscopic gastric band and sleeve gastrectomy) seem to progress with higher food intolerance when compared to procedures such as malabsortive biliopancreatic diversion associated with duodenal switch or mixed techniques, such as Roux-en-Y gastric bypass (RYGB)^18^.

Food intolerance after restrictive techniques tends to improve over time. However, if intolerance persists, the patient may experience severe nutritional deficiencies and commit to weight loss. It must remember that in the presence of behavioral disorders (anxiety and binge eating, so frequent in obese^9^^,^ 11), it is necessary interdisciplinary assistance that can better feed adaptation in postoperative^4^.

To specifically assess the food tolerance Suter et al.^20^ proposed a specific questionnaire called "Quality of Alimentation." It consists of four parts: 1) overview of patients satisfaction about their food quality; 2) the time between meals and food intake between them; 3) evaluation of the tolerance of eight different types of food; and 4) evaluating the frequency of vomiting or regurgitation. Score is obtained by evaluation of parts 1, 3 and 4 of the questionnaire. Part 1 refers to the general satisfaction as to how one can eat, and presents answers ranging from "excellent" to "very bad". In Part 3, the question refers to the ability to be able to eat certain types of food without difficulty, with some difficulty or not able to eat. In part 4, the question refers to the frequency of vomiting/regurgitation, with scores ranging from 0 to 6. Part 2 does not enter the score and have questions relating to the main food of the day, how many meals are made daily and the patient is fed between them. The score can range from 1 to 27, 27 being the highest score, referring to the excellent food tolerance.

For validation, questionnaire was administered to 300 patients who underwent gastric banding (BG) and 600 who underwent RYGB. It was also applied in a group of 75 non-obese patients for validation in the normal population, and a group of 55 morbidly obese patients not operated. The authors concluded that the questionnaire was simple to be answered (taking less than two minutes), easy reproducibility and allowing easy comparison between the individuals^20^.

This study was conducted due to the scarcity of publications on the subject of food tolerance in post-bariatric surgery and the importance of better understanding how the surgical techniques differ as to this issue.

The objective therefore was to conduct a systematic review of the literature on studies that used the questionnaire "Quality of Alimentation" to assess food tolerance in patients undergoing bariatric surgery and compare results in different surgical techniques.

## METHOD

This is a systematic review using the periodic portals Medical Literature Analysis and Retrieval System Online (Medline) and Scientific Electronic Library Online (SciELO).

In June 2015, it was accessed the portals using descriptors in English, in the following combinations: "Quality of alimentation, Food tolerance and bariatric surgery", "Food tolerance and Suter", "Food tolerance and bariatric surgery". Also references were searched using the key-words in Portuguese and Spanish, in the same combinations. Were sought only studies published since 2007, the year of publication of the article concerning the questionnaire "Quality of Alimentation." The search resulted in 90 references from Medline and four in Scielo. After exclusion of six repetitions, was proceeded reading the abstracts of the remaining references and, after excluding those not using the proposed questionnaire, were obtained 14 articles, 13 of Medline and one in Scielo. Thus, there was a complete reading of 14 studies, which formed the basis of this paper.

The 14 articles were classified according to the following variables: authors, year of publication, where the study was conducted, sample characteristics, type of study, research objectives, data collection method, and key findings.

## RESULTS

Firstly was sought the name of the first author, year of publication and where the survey was conducted, appearing on the first page of articles. Then, the characteristics of the sample, which the intervention used, type of research (prospective or retrospective), the objectives as described by the authors, the details of the procedures for data collection and the main conclusions. The details of the characteristics of the analyzed articles are shown in Table 1.

Of the 14 articles, 12 were published in English and two in Spanish. In addition, 10 articles were prospective and four retrospective. As for sampling, the total of 2745 patients were interviewed between 14 selected articles. Of these, 371 were submitted to BG, 1006 the vertical gastrectomy (GV), 1113 to RYGB, 14 to biliopancreatic diversion associated with duodenal switch, 83 were not operated or obese before surgery, and 158 were non-obese patients. Table 2 shows this distribution.

**TABLE 1:** - Distribution of published articles that used the questionnaire "Quality of Alimentation" and their characteristics

**Authors, publication year and place where it was held**	**Sample**	**Study type**	**Objectives**	**Collect data**	**Conclusion**
Suter et al. 2007, Switzerland	300 patients after BG, 600 after RYGB, 75 non-obese and 55 non-operated obese	Prospective	Develop a questionnaire to assess food tolerance in the postoperative follow-up	Quarterly during the 1^st^ year after surgery, every two years from 2 to 5 years after surgery and annually thereafter	Patients after RYGB had better food tolerance over time compared to BG; the questionnaire is reliable, easy to use and comfortable to the patient
Schweiger et al., 2010, Israel	218 patients (99 after RYGB, laparoscopic adjustable BG 49 after, 56 after GV and 14 after biliopancreatic diversion with duodenal switch)	Prospective	Evaluate food intolerance and food quality compared to surgical technique and postoperative time	Patients divided into 3 groups according to postoperative: 3-6 months, 6-12 months and longer than 12 months; questionnaire administered once in each group	Patients after laparoscopic adjustable BG had lower quality on food compared to other techniques evaluated by the study
D'Hont et al., 2011, Belgium	83 patients after laparoscopic GV and 83 non-obese	Retrospective	Compare the group undergoing laparoscopic GV with the non-obese	Postoperatively GV laparoscopic and is not specified at what time	The tolerance was significantly higher in non-obese patients
Keren et al., 2011, Israel	119 patients after laparoscopic GV, 83 regularly followed postoperatively and 36 irregularly	Retrospective	Compare the two post-surgical groups GV laparoscopic	In both groups to complete 30 months of postoperative follow-up	The group that showed regular consultations at the clinic showed better food tolerance
Romy et al., 2012, Switzerland	442 patients (221 ​​and 221 after GV and RYGB)	Prospective	Compare the two surgical techniques	At 1, 2, 3, 4 and 5 years postoperatively	The food had better tolerance and remained unchanged after RYGB
Ramon et al., 2011, Spain	105 patients, 64 underwent GV and 41 gastric bypass	Prospective	Assess the impact of GV and gastric bypass in food quality	Preoperatively and 3, 6, 12 and 24 months postoperatively	The food quality has worsened in the first months after surgery, improving gradually. There were no differences between the techniques evaluated
Overs et al., 2012, Australia	129 patients (13 after adjustable BG, 41 after RYGB, after GV 62 and 14 pre-surgical obese patients)	Prospective	Investigate and compare food tolerance after adjustable BG, RYGB and GV	Between 2-4 years of postoperative	The control group (non-operated obese) had better food tolerance. Adjustable patients after BG had lower food tolerance than the other techniques evaluated
Godoy et al., 2012, Brazil	47 patients after RYGB	Prospective	Investigate the level of food tolerance after RYGB	Average 2 years postoperatively	Patients with lower socioeconomic status have shown significant worst food tolerance
Sioka et al. 2013 Greece	110 patients after GV	Prospective	To evaluate the dietary profile after laparoscopic GV	Group 1 (postoperative <3 months), group 2 (3-6 months), group 3 (6-12 months), Group 4 (1-2 years), Group 5 (2-3 years) and 6 group ( > 3 years)	Improved food tolerance after the first year of laparoscopic GV
Keren et al., 2014 Israel	114 patients after laparoscopic GV	Retrospective	Evaluate the long-term food tolerance after laparoscopic GV	At 30 and 60 months postoperatively	Food tolerance at 30 months was better than at 60 months, with no significant difference
Kafri et al. 2013 Israel	37 patients (12 GV revised after laparoscopic and 25 after primary laparoscopic GV)	Prospective	Investigate food tolerance between the two groups	Average 18 months postoperatively in both groups	Food tolerance was lower in laparoscopic GV revised
Freeman et al., 2014, Australia	130 patients (14 pre-surgical obese controls, adjustable BG 13 after, 62 after GV and 41 after RYGB)	Prospective	Evaluate food tolerance between adjustable BG, GV and RYGB	Between 2-4 years of post-operative	Positive association between food intolerance and diet quality, low food tolerance considered as post-surgical complication after adjustable BG compared to GV and RYGB
Kafri et al., 2011, Israel	60 patients after laparoscopic GV	Retrospective	Evaluate food tolerance in two postoperative moments	Group 1 over a year of monitoring and Group 2 less than one year	Significant improvement of food tolerance over time
Acosta et al., 2010, Venezuela	41 patients (23 after RYGB and 18 after laparoscopic GV)	Prospective	To evaluate changes in quality of life and tolerance to food after bariatric surgery	Preoperatively and 3.6 and 9 months postoperatively	Worsening of tolerance at 3 months postoperatively, improving over time

BG=gastric band; BGYR=Roux-em-Y gastric bypass; GV=sleeve gastrectomy

**TABLE 2:** - Distribution of the initial sample of research subjects with and without operation, which evaluated the food tolerance by the questionnaire "Quality of Alimentation"

**Participants**	**Surgical technique**	**n**	**(%)**	
With bariatric surgery	BG	371	13,6	
	GV	1006	36,6	
	BGYR	1113	40,5	
	Biliopancreatic diversion associated with duodenal switch	14	0,5	
Without operation	Obese	-	83	3
	Non-obeso	-	158	5,8
Total			2745	100

BG=gastric band; GV=sleeve gastrectomy; BGYR=Roux-en-Y gastric bypass

## DISCUSSION

The questionnaire "Quality of Alimentation" was validated by Suter et al.^20^ in 2007 and is used in 300 patients undergoing BG and 600 RYGB, as well as validation in control groups. The authors observed that after BG, food tolerance was considerably reduced compared to the normal population. The RYGB, in turn, decreased the tolerance in the first year after surgery, especially in the first half due to postoperative edema, but returned to normal thereafter. When comparing the techniques, it was noted (except in the first quarter) that food tolerance in RYGB was significantly much better than in BG. This pioneering study has shown that the quality of food has decreased in both techniques, but improved over time and is affected by the type of chosen procedure.

Studies comparing BG with any other surgical technique came to the conclusion that it is the procedure with poor food tolerance in postoperative^3^^,^ 15 ^,^ 18 ^,^ 20. Because it is purely restrictive technique, this result was expected. Several authors have formulated hypotheses to explain the poor performance of the band. It could be adjusted, worsening the level of food tolerance. Possible inflammation due to foreign material and complications such as erosion or collapse of the bandage could also explain these results^15^^,^ 18 ^,^ 20.

It is reported that up to one third of cases of removal of the band is allocated to persistent food intolerance, even after its total emptying^15^. In Brazil, this is one of the least used techniques for weight loss by surgeons.

A study^3^ correlated food tolerance and quality of the diet. In BG, whose procedure had the worst tolerance, it was also demonstrated poorer diet quality. Associated with this, the patients ended up having less weight loss when compared to other techniques evaluated (GV and RYGB). This is due to the low feeding tolerance, which "force" patients to ingest high-calorie semi-liquid foods such as, for example, condensed milk, ice cream and chocolate.

 Comparisons between RYGB and GV, mixed and restrictive techniques, respectively, have shown controversial results in literature^1^^,^ 3 ^,^ 15 ^-^ 18. Some reports improved food tolerance in mixed technique, as expected^17^^,^ 18. Others showed that GV showed better food tolerance in postoperative^1^^,^ 3 ^,^ 15. Yet another study found no difference between any of these techniques^16^. These controversies in the literature can be justified by several variables that could have changed the results, such as longer follow-up with a greater chance of gastrointestinal adaptation and hence greater tolerance; inclusion of a control group for comparison with the patients who underwent the operation; significant sample size for correct statistical evaluation and subjective score of the questionnaire (part 1) with high results due to satisfaction with weight loss and not the food itself. In view of these variables it's hard to really establish which of these two techniques results in better food tolerance; more studies in this area are needed.

Food tolerance after using the biliopancreatic diversion technique associated with duodenal switch was the one that had better food tolerance when compared to BG, GV and RYGB. This result supports the hypothesis that disabsorptive or mixed procedures end up having better alimentary tolerance^18^.

Studies^6^^-^ 7 ^,^ 19 specifically evaluated whether the time of postoperative follow-up on the GV influenced food tolerance. The literature has demonstrated that with the passage of time tends to improve^6^^,^ 19 feeding tolerance, not only in the GV technique, but also in other 18 ^,^ 20. Among all the articles, one of them did not observe significant differences in food tolerance among patients undergoing laparoscopic GV, at 30 and 60 months after^7^. Perhaps this finding can be explained by the small weight loss the study population, since 82 of the 114 patients did not reach 50% loss of excess weight at 60 months postoperatively. This could influence the subjective score of the questionnaire, as previously mentioned.

In a study of specific assessment of food tolerance in non-obese patients and in patients undergoing GV, it was observed that food tolerance was better in the pre-operative^2^, as well as everyone else that compare to a control group non-operated^3^^,^ 15 ^,^ 20, realizing that food tolerance is better preoperatively. However, in RYGB late postoperative is possible to have the same food tolerance than controls^20^, lacking long-term studies to assess food tolerance before and after surgery.

It is of great importance to active participation by patients postoperatively. It has been shown that after 30 months of follow-up, patients who underwent laparoscopic GV participating actively, went regularly to consultation and had access to multi-professional team, had better food tolerance than the group that did not participate^8^. A study in Brazil showed the importance of the multidisciplinary team; the Brazilian Society of Bariatric and Metabolic Surgery recommends this staff to care patients with obesity. With it, it is easier to perform specific optimization for food tolerance, with better degree of adaptation after surgery with the recommendations and correct monitoring^4^. This same paper also demonstrated that low educational level is associated with low food tolerance after surgery, most likely due to poor adherence in follow-up visits.

Comparing the laparoscopic GV primary done and GV as revision procedure made after failure of adjustable BG, was found something interesting. Food tolerance was significantly lower in the second group, as well as higher frequency of vomiting. With these results it is necessary to develop specific pre- and post-surgical treatment to promote better behavioral results for the increasing number of patients undergoing to repeat bariatric surgery^5^.

The questionnaire "Quality of Alimentation" is simple and fast to be filled, but some conditions on its implementation are needed. The part 1 has influence on the comparison of techniques RYGB and GV, as the subjective component interfere in the final score. Part 2 of the questionnaire does not enter in final score and in most of the analyzed studies, was not used. Another factor was related to the questionnaire name. Articles in English, for the most part, use the term FTS (Food Tolerance Score) to refer to the questionnaire. As for the articles in Spanish use the term Calidad de la Alimentación. Ideally the use of standardized questionnaire original name (Quality of Alimentation) with their translations should be recommended. This would facilitate the search for publications about its use and avoid confusion in interpretation.

However, more research needs to be carried out comparing the usual surgical techniques in long-term follow-up.

## CONCLUSION

The questionnaire "Quality of Alimentation" is fast and easy way to evaluate food tolerance in patients subjected to various techniques of bariatric surgery. The biliopancreatic diversion with duodenal switch has the best food tolerance postoperatively compared to vertical gastrectomy and Roux-en-Y gastric bypass. As for gastric banding, there is still controversy with its use, since it showed the worst results.
